# Impacts of Microplastics on the Early Life Stages of Fish: Sources, Mechanisms, Ecological Consequences, and Mitigation Strategies

**DOI:** 10.3390/toxics14010027

**Published:** 2025-12-26

**Authors:** Imran Ullah, Haotian Chen, Jun Wang, Hashmi Kaiser, Abdallah A. Basher, Jiajia Li, Xuexia Zhu

**Affiliations:** 1 College of Animal Science and Technology, Yangzhou University, Yangzhou 225009, China; imranfashakhan77@gmail.com (I.U.); chenht241223@163.com (H.C.); junwang1990@yzu.edu.cn (J.W.); anupamhashmi@gmail.com (H.K.); hammodavet@gmail.com (A.A.B.); 2 Key Laboratory of Genetic Breeding and Cultivation for Freshwater Crustacean, Ministry of Agriculture and Rural Affairs, Freshwater Fisheries Research Institute of Jiangsu Province, Nanjing 210017, China; 3 College of Oceanography, Hohai University, 1 Xikang Road, Nanjing 210098, China

**Keywords:** aquatic ecosystems, microplastic, developmental abnormality, physiological stress, bioaccumulation

## Abstract

Microplastics represent an emerging threat to aquatic environments and organisms, as they infiltrate water systems, are ingested by marine species, and cause physical harm, endocrine disruption, and bioaccumulation up the food chain, potentially impacting biodiversity and human health. Aquatic ecosystems face considerable harm from microplastic pollution because fish in the early developmental stages, including embryos, larvae, and juveniles, are more susceptible due to their immature physiological and detoxification systems. This review aims to comprehensively explore the impacts of microplastics on the early life stages of fish. Aquatic environments receive primary and secondary MPs from urban runoff and industrial waste, together with degraded plastics, which affect fish embryos and larvae via direct ingestion, surface adhesion, and trophic transmission pathways. The physical impact of MPs causes digestive tract blockages that reduce hatching success and create developmental problems in fish organs, but chemical toxicity develops from plasticizers, heavy metal leaching, and pollutant adsorption, which causes oxidative stress, endocrine disruption, and metabolic dysfunction. Survival rates decrease because exposure causes fish to perform poorly during swimming activities and make limited efforts to avoid predators. The small dimensions and high chemical reactivity of MPs increase their bioavailability, which promotes tissue penetration and leads to accumulation at different levels of the food chain. This comprehensive review emphasizes that we need to establish uniform detection protocols, long-term exposure research, and effective strategies to control MP pollution. The resolution of these difficulties remains essential for protecting fish populations, as well as for protecting biodiversity and minimizing seafood contamination risks to human health.

## 1. Introduction

Microplastics (MPs) are defined as synthetic polymer particles and have emerged as a pervasive environmental pollutant, contaminating aquatic ecosystems worldwide [1]. According to ISO 21960:2020 [2], they are classified into two main groups: primary MPs, which are intentionally manufactured (e.g., cosmetic microbeads), and secondary MPs, which form through the natural degradation of larger plastic objects [1]. Particles smaller than 1 µm are classified as nanoplastics (NPs), whose diminutive size enables them to penetrate biological membranes and cellular barriers [3,4]. Three broader size ranges of plastic particles are commonly recognized: mesoplastics (500 µm to 5 mm), MPs (1 µm to 500 µm), and NPs (<1 µm) [5,6]. Plastics originate from fossil fuels (e.g., coal, natural gas, and crude oil) or renewable materials (e.g., cellulose, corn, and grains) [7,8]. Their durability, lightweight nature, ductility, and low cost have led to widespread use across industries, but these same properties contribute to their persistence in the environment [9]. MPs were first formally named by Thompson nearly two decades ago, marking the beginning of heightened awareness of their environmental impact [10,11]. Pioneering work by marine ecologist Richard Thompson in 2014 highlighted the pervasive spread of MPs in oceanic environments, demonstrating their accumulation in waterways worldwide [12].

Today, MPs contaminate all of Earth’s ecosystems, including marine and freshwater bodies, terrestrial soils, the atmosphere, and even human tissues and fluids [13,14]. For instance, 21 types of MPs (20–500 µm) have been detected in human sputum, with polyurethane (PUR), polyester (PL), and chlorinated polyethylene (PE-C) predominating [15]. MPs have been detected in various human tissues and excretions, including stool [16], placentas [17], lungs [18], and breast milk [19], such as spherical or irregular shapes polypropylene (PP) MPs (5–10 µm) in the placenta, raising concerns about fetal development. Polyethylene terephthalate (PET), polyethylene (PE), and styrene polymers appear in human blood at concentrations up to 1.6 µg/mL, indicating systemic circulation [20]. Similarly, PP and PET fragments and films (50–500 µm) in human stool confirm ingestion and excretion [16]. Human exposure to MPs occurs primarily through contaminated food and drinkable water, including seafood such as fish, mussels, and crabs, as well as poultry and edible plants via the food chain [21]. Global plastic production and disposal have exacerbated environmental damage. In 2022, the world produced 400.3 million tons of plastics [22]. Without stronger policies, global plastic waste is projected to nearly triple to 1 billion tonnes by 2060, with mismanaged waste hitting 270 million tonnes [23]. Marine habitats contain 1–5 million tons of plastic litter, with oceans harboring over 5.25 trillion plastic particles weighing 268,940 tons [24,25,26]. Annually, 4.8–12.7 million tons of plastic enter oceans, constituting 60–80% of marine pollution [27]. The COVID-19 pandemic amplified this issue, with face mask production generating 698 million units of plastic waste in 2020 alone [28,29]. Escalating production and poor waste management have made plastic pollution a defining environmental threat of the 21st century [30,31]. Dietary exposure to MPs and NPs is now inevitable, as these particles infiltrate food systems, drinking water, and agricultural products through debris fragmentation and direct releases [32]. According to the United Nations Environment Programme, about 460 million tons of plastic are manufactured yearly worldwide, of which half are disposable. The amount of plastic garbage that ends up in rivers, lakes, and seas each year is around 8 million tons. This leads to an ongoing increase in the amount of micro- and nanoplastics (MNPs) released into the aquatic environment. Significantly, MPs have found their way into food chains, aquatic life, and even human organs, such as a newborn’s placenta. Their profound and wide-ranging effects have turned into a global environmental issue [33].

As ubiquitous environmental pollutants, MPs and NPs harm aquatic life and human health. In humans, MP accumulation elevates risks, including impaired male fertility and conception [34]. Their toxicity primarily induces oxidative stress, leading to cellular and DNA damage, which can progress to neurodegenerative diseases, reproductive issues, immunodeficiency, cell division impairments, structural damage, reduced cell survival, intestinal microbiome modifications, metabolic disturbances, dysbiosis, cancer, and respiratory conditions [35,36]. NPs pose an elevated risk due to their abundance, reactivity, and ability to cross biological barriers [37]. Over six decades of plastic ubiquity have drawn public, media, and scientific attention to these ecological and health consequences [38]. The fishery industry faces severe consequences from escalating global pollution, particularly in oceanic and freshwater environments, where MPs and other contaminants degrade aquatic ecosystems and threaten key exports like seafood [39,40,41]. MPs, increasingly detected in oceans, food chains, and atmospheres, harm fish health, reduce populations, and raise human consumption safety concerns, undermining economic stability for fisheries and dependent communities [40,41]. Compounding factors include rising ocean temperatures and acidification from climate change, prompting fish migrations that disrupt ecosystems and commercial operations [42]. Overfishing, habitat destruction, and pollution further deplete stocks amid growing demand, creating supply shortages and damaging production [43,44,45].

Fish in early developmental stages, such as embryos and larvae, are especially prone to microplastic pollution because of their fast-paced growth, nascent immune defenses, and restricted evasion capabilities, frequently resulting in particle ingestion or attachment that resembles natural food sources. This increased exposure triggers issues like stunted development, neurotoxic responses, weakened immunity, and higher mortality, which impair individual viability and escalate to population-level declines through diminished breeding success and lower recruitment. In turn, these shifts can unsettle aquatic ecosystems by altering food chains, eroding biodiversity, and straining fisheries reliant on stable fish stocks. This review synthesizes the current literature on MPs’ classification, sources, and stage-specific vulnerabilities in fish early life stages (i.e., embryo, larvae and juvenile), identifies crucial findings, and proposes mitigation strategies to support fishery sustainability and global environmental health. The aims of this review are to: (1) outline MP sources and exposure pathways relevant to fish early-life stages; (2) synthesize toxicity data with emphasis on stage-specific vulnerabilities; (3) explore comorbidities with other pollutants; and (4) propose research directions.

## 2. Classification of Microplastics

MPs are ubiquitous in both freshwater and marine ecosystems. Regarding polymer types, the most commonly reported in aquatic environments and ecotoxicity studies include polystyrene (PS), polyethylene (PE), polypropylene (PP), polyvinyl chloride (PVC), polyethylene terephthalate (PET), polyamide (PA, also known as nylon), acrylonitrile butadiene styrene (ABS), polyurethane (PU), and polytetrafluoroethylene (PTFE). This classification is essential for understanding their sources, distribution in aquatic environments, and impacts on organisms such as fish.

### 2.1. Primary Microplastics

**Figure 1:**
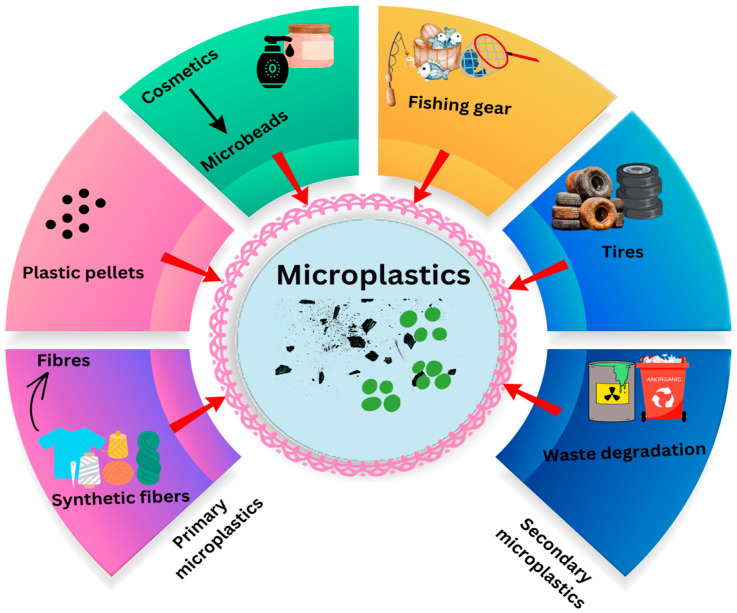
Showing different sources of primary and secondary MPs.

Primary MPs are intentionally manufactured small plastic particles, typically less than 5 mm, for use in consumer products and industrial applications [38,46,47]. They are incorporated into cosmetics, detergents, paints, pharmaceuticals, personal care products (e.g., toothpastes, facial scrubs, and diapers), and agricultural chemicals (e.g., pesticides and insecticides) to enhance texture, abrasiveness, and durability [31,48,49]. Manufacturers produce four main types: microbeads, pellets, granules, and spheres, each tailored to specific product functions (Figure 1) [50,51].

The textile and packaging industries are primary sources of these MPs (Figure 1) [52]. During laundering, synthetic fabrics release MP fibers (MPFs) into wastewater, with detergents exacerbating particle shedding [53]. Textile production processes from raw materials to finished products, involve fibers, yarns, beads, sequins, pearls, glitters, adhesive resins, and washing powders, all contributing to MP emissions [52,54,55]. The widespread use of primary MPs necessitates urgent regulatory measures and sustainable alternatives to curb their release into ecosystems.

### 2.2. Secondary Microplastics

Secondary MPs form unintentionally through the environmental degradation of larger plastic items via physical, chemical, and biological processes (Figure 1) [56]. Fragmentation occurs due to wave action, UV radiation, mechanical abrasion, and thermal oxidation, breaking down plastic waste into smaller particles [57,58]. Electron microscopy reveals their irregular, rough surfaces, distinguishing them from the smoother primary MPs [59]. Secondary MPs now dominate environmental pollution, surpassing other fragmentation products in prevalence [60].

Industrial waste, including plastic packaging, building materials, and durable goods, is the largest contributor to the secondary MPs (Figure 1) [61]. Of the approximately 370 million tons of plastic produced annually, only 9% is recycled, 12% is incinerated, and 79% ends up in landfills or the environment, serving as reservoirs for secondary MP formation [62] Sectors such as textiles, automotive, and construction further accelerate this fragmentation [63].

Secondary MPs exert profound effects on aquatic ecosystems, inflicting toxic damage on fish, oysters, mussels, and sea turtles by impairing immune and digestive systems, potentially leading to mortality [64,65]. A global study of 728 fish species revealed MP accumulation in gastrointestinal tracts, causing organ blockages, reduced feeding efficiency, stunted growth, and lowered survival rates [66,67]. As the predominant form of plastic pollution, secondary MPs threaten marine life and pose human health risks via seafood consumption. Mitigation requires innovative detection methods, prevention strategies, and responsive actions, including advanced wastewater treatment, regulatory enforcement, and technological innovations to halt their entry into aquatic systems [68,69].

### 2.3. Methods for Identifying Microplastics

Identifying MPs is essential for evaluating their environmental prevalence and toxicity, yet it poses significant methodological challenges. Common identification techniques include visual microscopy and stereomicroscopy for initial size and shape classification of particles larger than 50 μm, though these methods suffer from subjective bias in particle selection and fail to confirm polymer composition or detect impurities [70]; Fourier Transform Infrared Spectroscopy (FTIR), which identifies polymer types through infrared absorption spectra and enables analysis of particles down to 10–20 μm via micro-FTIR, but faces challenges from spectral interference caused by adhered organic matter, additives, or monomers that obscure signals, necessitating rigorous sample cleaning such as hydrogen peroxide digestion, while impurities like phthalates or bisphenol A can lead to overlapping peaks and misidentification [71]; Raman spectroscopy, which complements FTIR for particles as small as 1 μm by using laser-induced vibrational spectra, yet is hindered by fluorescence from dyes or impurities in colored plastics that can overwhelm signals, alongside long acquisition times that elevate analysis costs [72]; and pyrolysis-gas chromatography-mass spectrometry (Py-GC/MS), which quantifies polymer composition and detects additives or monomers through thermal decomposition, although high temperatures may alter impurity profiles and its sensitivity is limited for trace impurities in complex environmental matrices [73]. NPs pose unique challenges due to their small size, high surface-to-volume ratio, and aggregation tendency, complicating collection, identification, and toxicity assessment [74]. Identification demands advanced techniques like dynamic light scattering (DLS), NP tracking analysis (NTA), transmission electron microscopy (TEM), or atomic force microscopy (AFM), requiring high-purity standards to differentiate NPs from natural colloids [75]. These issues impede field quantification, underestimating NP risks in aquatic ecosystems where barrier penetration heightens ecological and health threats.

Concentrations of plastic particles are reported diversely across research groups, with common formats including number-based metrics (e.g., particles/m^3^ or items/L), mass-based units (e.g., mg/L or μg/g), volume-based (e.g., % volume), and surface area-based (e.g., items/km^3^). These variations arise from differences in sampling techniques (e.g., plankton nets vs. pumps), size thresholds, environmental matrices, and analytical goals [76,77]. To enhance comparability, standardized frameworks like those from GESAMP or ISO are recommended, promoting hybrid metrics (e.g., combining number and mass) for comprehensive evaluations [78].

## 3. Sources of Microplastics

MPs originate from diverse land- and sea-based activities, entering aquatic environments through direct release, degradation, or transport. For early-life stages of fish, sources that contaminate shallow nursery habitats, spawning grounds, and water columns are most relevant, as these stages have limited mobility and higher susceptibility to ingestion, adhesion, or maternal transfer. Key priorities include urban runoff and wastewater effluents, industrial discharges from textiles, consumer products, and marine debris from fishing gear. Identifying and prioritizing these stage-relevant sources is essential for mitigating contamination and assessing risks to fish populations.

### 3.1. Urban Runoff

Urban runoff acts as a primary pathway for MPs into aquatic systems, mobilizing particles from human activities during rainfall [26,79]. Sources include degraded plastic litter forming secondary MPs via UV radiation and abrasion [80], tire and road wear particles (TRWP) from vehicles accumulating on pavements [79,81] and microfibers from synthetic textiles released during laundering, which evade treatment via combined sewer overflows (CSOs) or settle before rain washes them away [82]. Impermeable surfaces (e.g., roads, rooftops) accelerate flow and pollutant collection, with ~70% of stormwater infrastructure discharging unfiltered runoff into water bodies [83,84]. Heavy rains trigger CSOs, releasing untreated sewage and MPs [85]. Sub-optimal constructed wetlands can become MP reservoirs [86], but optimized bioretention systems effectively reduce MPs, nitrogen, and particulates through filtration and microbial processes.

Quantitative data underscores runoff’s impact: TRWPs contribute 42% to European river MPs, storm-water accounts for 43% in Germany’s Warnow estuary, and 62% of Baltic Sea MPs stem from runoff and overflows [65,66,67,68]. Runoff mixes precipitation with sediments, debris, heavy metals, organic pollutants (e.g., pesticides, PAHs), nutrients, and MP [87,88]. Urban density amplifies production via tire wear, landfills, sewage, construction, industry, transportation, and household laundry [89]. MPs lightweight properties enable cross-media mobility (air, dust, soil, water), with storm-water channeling land-based MPs to rivers and coasts [90]. Additional inputs occur via sewage and recreational activities (e.g., watersports, fishing) [91].

Runoff also carries emerging contaminants like MPs and phthalate acid esters (PAEs), harming aquatic life and humans [92]. Street accumulation of plastics and TWPs contaminates rivers, lakes, and reservoirs, alongside agricultural and industrial sources [88,93]. Mitigation starts with source identification and pathway control for sustainable water protection, including enhanced bioretention and filtration. Further studies are needed to quantify runoff’s role and refine strategies [94].

### 3.2. Industrial Waste

Industrial processes are key MP contributors, with contamination levels linked to development intensity [95]. Primary MPs form during virgin plastic production, where monomers are granulated for transport and melted into products like chairs, packaging, bottles, and containers [96]. Post-production waste (defectives, unused materials) enters recycling or the environment [21]. Non-degradable plastics persist long-term, threatening ecosystems and health [97]. Particles < 5 mm in products (e.g., soaps, washes, pharmaceuticals, scrubs) evade detection and removal [98].

The textile sector is a major source, releasing microfibers from synthetics (e.g., PL, nylon, acrylic) during manufacturing and washing [97]. Waste includes pre-consumer (production scraps), post-consumer (discarded clothing), and commercial (packaging) [99]. Microfibers bypass wastewater filters, reaching rivers and oceans [100,101], especially in textile-heavy regions with inadequate treatment [52]. Synthetic industries emit high MP volumes, spreading across atmospheres, soils, freshwaters, and seas [102]. Non-biodegradable materials degrade into persistent fragments, exacerbating pollution [103]. Effective control requires improved recycling, waste management, and regulations to reduce aquatic releases and protect fish.

### 3.3. Consumer Products

Consumer goods release MPs via use and degradation, posing analytical challenges. In bottled water, MP concentrations vary widely due to methodological and instrumental differences [104,105]. Accurate measurement is critical for evaluating abundance, transport, and degradation of packaging plastics (e.g., PE, PP) under environmental factors [106,107].

The COVID-19 pandemic amplified pollution through surged personal protective equipment (PPE) demand, generating polypropylene waste from masks and gloves via improper disposal [61,108]. Used masks release thousands of microfibers during disinfection [109]. MPs serve as vectors for contaminants (e.g., heavy metals, PAHs, organics), heightening health impacts [110]. Single-use items (e.g., cups, PET bottles, packaging, cutlery) generate post-consumer waste that degrades into persistent MPs, dispersing through water, soil, and air [110,111]. Addressing this demands usage analysis, policies, and public initiatives to curb releases, safeguarding ecosystems and health [61,104,110].

### 3.4. Marine Debris

Marine debris encompasses human-origin solid materials entering oceans via rivers, wastewater, wind, or direct disposal [111]. Land-based sources contribute 80%, with sea-based activities (e.g., fishing) adding 20%; plastics comprise 60–95% of floating litter [112,113]. Exponential production, poor management, and degradation resistance make plastics a severe threat [114].

The entry of waste/debris into the ocean and beaches is influenced by several factors such as weather and tidal patterns, as well as proximity to urban, industrial, and recreational areas, seaways, and fishing areas [115]. Plastics affect at least 267 marine species, originating from land activities and accumulating as debris [116]. There have been reports of plastic waste in marine and coastal habitats for at least 45 years, showing it is chronically present [115]. Debris includes metals, rubber, glass, paper, plastics, and textiles, with plastics dominating due to inadequate land management [117,118]. Improperly handled waste enters via sewage, rivers, and wind, threatening all ocean compartments, species, ecosystems, and economies [119]. There are currently over 8300 million metric tons of plastic in the environment, most of which is permanently marine debris [120].

Debris origins include ocean-based (e.g., fishing nets, ropes, buoys, gloves, plastic sheets) [121], land-based (e.g., syringes, beverage cans, straws, cotton swabs, tampon applicators) [122], and general (unidentified plastics) [109]. Entry points involve improper disposal, accidental losses, and natural disasters. Assessment methods (i.e., visual surveys, net trawls, and diving) quantify types, quantities, and distributions to inform solutions [123].

Impacts include entanglement, habitat destruction, and toxicant transport (e.g., heavy metals, POPs adhering to MPs), harming marine life and entering food webs [124]. Solutions demand international regulations on waste and production, reduced single-use plastics, community cleanups, and education to curb ecological and economic damage [113,120]. Linking sources, distribution, and consequences enables effective strategies for marine ecosystem protection [112,120].

## 4. Pathways, Concentrations, and Hotspots of Microplastics in Aquatic Ecosystems

### 4.1. Primary Pathways of MPs Entry

Primary pathways include improper waste management, river transport of degraded larger plastics, urban runoff, stormwater drainage, industrial discharges, and tourism-related litter. In marine systems, other routes involve atmospheric deposition, accumulation in wastewater treatment inefficiencies and environmental transport [118,125,126]. Ultimately, Fluvial channels are significant delivery pathways of MP pollutants to the bigger aquatic systems like as the seas [127]. Freshwater sources similarly encompass consumer product pollution (e.g., microbeads), land-based disposal, and biological transfer by aquatic organisms, which ingest and redistribute MPs across ecosystems [128]. Uncontrolled land disposal serves as a major entry point, with rainfall washing MPs from urban areas into rivers and seas, exacerbating spread [128]. Microfibers and other particles can be deposited by the atmosphere and land directly on water surfaces. As convergent routes, tributary streams gather MPs from all over a watershed and carry them into the main river channels [127].

### 4.2. Stage-Specific Exposure Routes, Bodily Presence, and Concentration Variations in Fish

These pathways intersect with fish early-life stages in habitat-specific ways, influencing exposure routes, bodily presence, and concentration variations. In embryonic stages, exposure primarily occurs via maternal transfer or direct adhesion to the permeable chorion, leading to aggregation on the egg surface rather than internal penetration, which can impair hatching and development. Larval stages face heightened risks through ingestion, as MPs mimic zooplankton prey, resulting in gut accumulation that causes physical blockages, reduced feeding, and bioaccumulation of associated toxins; concentrations in larvae can be higher due to their small size and high feeding rates, with studies showing ingestion even in yolk-sac stages before active feeding. Juvenile fish exhibit varied exposure via ingestion and gill uptake during foraging, leading to tissue distribution (e.g., in guts, livers, and muscles) and behavioral changes; MP concentrations may decrease relative to larvae but persist, with species-specific uptake influenced by habitat and diet [129,130,131].

### 4.3. Concentrations of Microplastics in Aquatic Ecosystems

Freshwater ecosystems generally exhibit higher MP concentrations than marine environments, with surface water levels ranging from 0.028–1146 items/m^3^ and sediments from 1.20–616.1 items/kg, compared to marine ranges of 0.02–102,550 items/m^3^ in water and 3.00–390.7 items/kg in sediments [132,133]. Average densities further highlight this disparity, with freshwater bodies at 1.8–2.4 pieces/L versus 0.9 pieces/L in marine settings [132]. Such concentrations in freshwater highlight the necessity of additional study of the long-term ecological implications on species, especially typical hotspots such as estuaries and coastal areas where the concentrations are higher and fish developmental stages in their early stages are more abundant [134]. Variations in MP concentrations across stages are evident in these hotspots; for instance, larval fish in estuarine nurseries may encounter elevated levels (up to several items per individual) due to particle settling, while embryonic exposures in freshwater spawning grounds show lower internal concentrations but higher surface adhesion.

### 4.4. Microplastic Hotspots and Implications for Fish Early-Life Stages

MP hotspot areas with elevated pollution levels, often overlapping with ecologically vital regions and high human activity, pose severe threats to marine biodiversity, especially fish early life stages. Near-shore habitats, critical as nursery grounds for species like juvenile seabream, expose developing fish to high plastic contaminants, while larvae ingest MPs as early as the yolk-sac stage, even before active feeding [95,129,135,136]. Prioritizing research and monitoring in these zones is essential for informing conservation, contamination prevention, and public health strategies. Estuaries, where freshwater rivers mix with ocean water, accumulate MPs due to river runoff, discharged waste, and stormwater inputs, as evidenced in the Yangtze River estuary with significant levels harming aquatic life [134]. These biodiversity hotspots support crucial life stages of many fish species, making them priority sites for studying MP distribution and effects on marine ecosystems. Coastal zones emerge as key MP hotspots due to converging human activities (e.g., pollution sources), ocean currents, and biological factors that increase particle density and mass transport [134]. Serving as habitats for diverse marine species, these dynamic areas collect substantial MPs, endangering early life stages and amplifying ecological risks [137].

## 5. Vulnerability of Early Life Stages of Fish to Microplastics Exposure

Early life stages of fish—embryos, larvae, and juveniles—are particularly susceptible to MP pollution, as evidenced by numerous studies showing negative population-level effects [3,25,28,54,121,122]. These stages exhibit heightened vulnerability due to fragile physiological systems, permeable barriers, rapid metabolism, and large surface area-to-volume ratios, which amplify exposure to MPs physical and chemical impacts. Consequences include developmental impairments, hatching failures, stress-induced mortality, and behavioral changes, all of which reduce survival rates and threaten aquatic ecosystems.

### 5.1. Embryo Stage

Fish eggs are highly vulnerable to MPs during embryogenesis, with species such as rainbow trout (*Oncorhynchus mykiss*), zebrafish (*Danio rerio*), yellow catfish (*Pelteobagrus fulvidraco*) and marine medaka (*Oryzias melastigma*) displaying developmental defects, as shown in Table 1 [138,139,140,141,142,143]. MPs, including microfibers, polystyrene (PS), and polyethylene (PE) particles, adhere to egg surfaces or penetrate the chorion, causing retarded maturation, reduced hatchability, growth retardation, and cardiac abnormalities (e.g., arrhythmia, decreased output) that lower post-hatching survival [117]. For instance, PS MPs on zebrafish (*D. rerio*) chorions block oxygen transport, slowing development and hatch rates [144], while PE MPs in marine medaka (*O. melastigma*) embryos induce heart issues [145]. Additionally, MPs could threaten wild egg survival by decreasing buoyancy, increasing predator vulnerability, and facilitating pollutant uptake [146].

Stereoscopic microscopy of 349 catfish eggs (200–300 μm) revealed microfibers in 25 samples, comprising 50% rayon, 30% PET, and 20% natural cotton, often pigmented in blue, black, or red [123]. This underscores widespread water pollution affecting embryonic stages. In marine medaka (*O. melastigma*), adult exposure to PS MPs reduced reproductive capacity, delaying oocyte maturation and yielding offspring with slower heart rates and shorter body lengths [147]. Rainbow trout (*O. mykiss*) eggs encounter MPs immediately, with particles attaching externally or breaching the chorion based on size [148]. Internal uptake causes developmental issues, reduced heart rates, metabolic slowdowns, oxidative stress, disrupted cellular division, and endocrine interference from adsorbed pollutants (e.g., phthalates, bisphenols) [149,150,151,152,153]. Post-hatching, exposed embryos exhibit altered feeding and swimming behaviors, diminishing natural survival [154]. These findings highlight the urgent need to control MP pollution, as egg penetration disrupts embryonic metabolism, behavior, and ecosystem health.

Female reproduction is also impacted, with acute PS-MP exposure increasing gravid females but reducing egg numbers and fertilization rates, suggesting ovarian dysfunction [155]. Furthermore, PS fragments disrupt embryonic development and bloodstream function [156], with zebrafish (*D. rerio*) chorion encapsulation causing hypoxia and death [141]. Exposure to 46 nm PS-NPs (2 mg/L, 21 days) reduced egg counts without altering ovarian index, whereas exposure to 5.8 μm PS-MPs under identical conditions did not produce significant changes in either egg production or ovarian index [157]. Grass carp (*Ctenopharyngodon idella*) embryos at 45 mg/L MPs showed no survival or hatching impacts, It shows that this species has a very high tolerance threshold for early life stages, indicating that, at least at environmentally relevant concentrations below this threshold, acute toxicity from MPs alone might not be the main risk factor for grass carp (*C. idella*) embryonic development, but exhibited deformed, wrinkled membranes via SEM [158]. Cuttlefish (*Sepia officinalis*) yolk analyses via Raman microspectroscopy detected MPs < 5 μm, with higher concentrations in yolk than embryos [159]. Butter catfish (*Ompok bimaculatus*) and striped dwarf catfish (*Mystus vittatus*) eggs contained 0.0087 ± 0.029 and 0.0078 ± 0.027 MPs/g, respectively, while climbing perch (*Anabas testudineus*) and Stinging catfish (*Heteropneustes fossilis*) showed none [160].

**Table 1:** Summary of toxicological effects of MPs on embryonic stages of various fish species.

Fish Species	MPs size	MPs Concentration	Exposure Time	Effects	Ref.
zebrafish (*D. rerio*)	PS, 1–3 μm	0.01–10 mg/L	3 days	Increased heart rate, oxidative stress, apoptosis	[161]
	PS, 100 μm	3.84 × 10^−8^ g/mL	4 days	Pigmentation deficiency and head region malformations; no mortality	[162]
	PS, 157 ± 52 μm	250-items/50 mL	3 days	Reduced hatching of the embryos, toxicity, hypotoxicity	[163]
marine medaka (*O. melastigma*)	PS, 10 μm	2, 20,and 200 μg/L	28 days	Decreased hatching rate, increased developmental abnormalities, suppressed growth	[164]
	PE, 4–6 µm	0.01–16.64 μg/L	12 days	Significantly reduced embryonic survival (to 41.0%) and hatching rate, developmental deformities	[165]
rainbow trout (*O. mykiss*)	PS, 300 μm	environmentally realistic concentrations	69 days	Reduced hatching rate, embryo toxicity, increased genotoxicity endpoints in PS pellet treatment	[166]
bighead carp (*Hypophthalmichthys nobilis*)	PS, 5 µm	0.5, 5, and 50 mg/L	2 days	Accelerated Hatching, high embryo mortality shortly after hatching, all embryos/larvae in five of the experimental groups died	[167]
fat greenling (*Hexagrammos otakii)*	PS, 10 μm	1 mg/L	27 days	Mortality rate increase, heart rate decrease, decrease hatching Rate	[168]
brown trout (*Salmo trutta*)	PET, 300 μm	700 mg/L	113 days	No change in hatching rate, minor variability in developmental time	[169]

### 5.2. Larval Stage

Seasonal sampling in Douro estuary (1498 larvae, density 11.66/100 m^3^) detected 2152 MP particles (17.06/100 m^3^), mostly fibers, fragments, and films associated with *Pomatoschistus* spp. and *Clupeidae*; MPs exceeded larval densities except in summer [170]. Fish larvae have been found to exhibit heightened sensitivity to MPs due to their small body size and underdeveloped morphology, which facilitate ingestion of size-matched MPs, causing gastrointestinal blockages, nutrient absorption issues, and mechanical damage (Table 2). Primitive, non-selective feeding increases the accumulation of MPs as they are mistaken for food. Immature detoxification and low metabolic reserves exacerbate impacts even at low concentrations, unlike in more resilient juveniles or adults. For instance, in zebrafish (*D. rerio*), valued as a model organism for its short lifecycle, low maintenance, and environmental sensitivity [171], PE-MPs ingested via the gastrointestinal tract caused gene expression changes within 48 h that normalized by 14 days without lasting abnormalities [172]. MPs disrupt gut microbiota and metabolomes of zebrafish (*D. rerio*) larvae, with PE’s properties not mitigating aquatic hazards [173,174]. Grunion larvae ingested MPs in controlled still-water (4 h) and turbulent (2 h) conditions. In still water, 4.76% of 147 larvae contained 9 particles in the gut, there were 7 and 3 immediate deaths from toxic and mechanical effects among 168 larvae (3–14 days old), and 7.7% ingested MPs, with 6 deaths; ingestion and mortality increased with concentration. Following a 2-h feeding trial in turbulent water, it was discovered that 9 larvae (6.30%) had a total of 24 visible MP particles in their digestive systems. After consuming MPs, two of the nine larvae had perished [175]. MP evaluation confirmed that Peled larvae were capable of ingesting 2 µm polystyrene (PS) microspheres under waterborne exposure conditions. A strong positive correlation (rs = 0.956; *p* < 0.01) was detected between MP concentrations in water and PS microsphere abundance in the gastrointestinal tract, with no significant differences between 24 h and 6-day exposure periods. MP ingestion induced significant alterations in digestive enzyme activities and whole-body antioxidant responses. Specifically, α-amylase and non-specific esterase activities were significantly elevated after 24 h of exposure (*p* < 0.05), whereas pancreatic trypsin, bile salt–activated lipase, and intestinal aminopeptidase N activities showed significant increases only after 6 days. Additionally, catalase activity was significantly enhanced following prolonged exposure [176].

### 5.3. Juvenile Stage

Juvenile fish exhibit high vulnerability to MPs, resulting in impaired growth, developmental complications, behavioral changes, and reduced ecosystem services. MPs can cause digestive issues, nutrient malabsorption, blood abnormalities, intestinal defects (e.g., epithelial damage, inflammation for particles < 10 μm), immune alterations, and histopathological changes in fish juveniles, with responses varying by species and influenced by diet and digestive structures (Table 3). In flounder juveniles collected from Le Havre Harbor and Canche estuary, 149 MPs (103 fibers, 43 fragments, 3 films) were detected in 86 individuals, showing contamination rates of 91.7% for caged fish (ingesting 75% of items in the 64–99.1% size range) and 36.4–80% for feral fish [186]. Zebrafish (*D. rerio*) juveniles exposed to fluorescent MP microbeads (1–5 μm and 40–47 μm at 50 mg/L) from larval to juvenile stages accumulated more small particles, enabling translocation to organs and disrupting growth and physiology [187]. Smaller MPs pose greater threats than larger ones. Schlegel’s black rockfish (*Sebastes schlegeli*) juveniles ingesting fragmented or fibrous PET showed increased apoptosis, phagocytic activity, immune gene expression, and hepatic metabolic changes after 72 h, with fibrous forms inducing stronger immunotoxic and cytotoxic effects via oxidative stress and reactive oxygen species (ROS) production [188,189]. Medaka juveniles exposed to PS MPs displayed no overt growth effects but exhibited histopathological modifications and reproductive delays, indicating subtle long-term impacts Juvenile tilapia (*Oreochromis niloticus*) (8–14 cm, 10–40 g) exposed to MPs for 30 days showed no mortality, health issues, or changes in feed intake (6.6–7.1 g/day) or production (7.6–8.3 g/day), with mean weights comparable across groups (26.2–27.5 g) [190].

Gilthead Seabream (*Sparus aurata*) juveniles fed PE-MPs (10–20 μm, 5 ± 1 μg/g fish/day via brine shrimp (*Artemia salina*) for 35 days) experienced higher mortality, altered brain and liver metabolites, and significant liver/intestine damage [191]. In largemouth bass (*Micropterus salmoides*), grass carp (*Ctenopharyngodon idella*), and common carp (*Cyprinus carpio*), MPs caused disordered/shortened intestinal folds and cellular infiltration in bass, but no significant vacuolization, goblet cell hyperplasia, villus shortening, or muscle thickness changes in common carp (*C. carpio*) [192]. Yellow catfish (*Pelteobagrus fulvidraco*) exposed to 500 ng/L oxytetracycline (OTC), low (100 μg/L) or high (1000 μg/L) PS-MPs, or combinations for 28 days showed no individual effects on growth, antioxidants (SOD, CAT), or digestive enzymes (trypsin, amylase, lipase), but low MP + OTC increased SOD/CAT, caused vacuolation, epithelial loss, and elevated *Proteobacteria* [140]. Kingfish (*Seriola lalandi*, <30 days post-hatch) juveniles ingested more biofouled MPs than clean ones, with increased swimming changes, as biofilm enhanced attraction [193]. Common carp (*C. carpio*) exposed to PE-MPs and 4-nonylphenol (4-NP) suffered histopathological damage (liver > gills > brain; severity: 4-NP > combination of 4-NP and PE-MPs > PE-MPs), with no recovery in high-toxicity groups [194]. Naturally aged MPs impaired growth, behavior, and health in zebrafish (*D. rerio*) juveniles, posing risks to freshwater species [195]. Overall, juvenile fish are highly vulnerable to MPs, which can cause growth retardation, hormonal imbalances, metabolic disruptions, oxidative stress, immunological problems, behavioral changes, and increased mortality.

**Table 3:** Summary of toxicological effects of MPs on juvenile stage of various fish species.

Fish Name	MP Size	MP Concentration	Exposure Time	Effect	Ref.
gilthead seabream (*S. aurata*)	PE, 10–20 µm	5 ± 1 µg/g fish/day	35 days	Increased mortality, liver and intestinal damage, altered brain and liver metabolites.	[189]
	PS, 1–20 μm	0, 25 and 250 mg/kg	21 days	Inflammation and immune alterations in intestine	[191]
	PVC MPs, 40–150 µm	100 and 500 mg/kg	30 days	Affect several vital organs; produce chronic stress	[196]
	Six MPs types	0.1 g/kg body weight/day	45 days	Not causing imminent harm to fish	[197]
yellow catfish (*P. fulvidraco*)	PS, 20 μm	0.115, and 1.5 μg/L	15 days	Effects of MPs (polystyrene) on specific growth rate (SGR), hypoxia-inducible factor-1α (HIF-1α), tumor necrosis factor-α (TNF-α), interleukin-8 (IL-8), and interferon (IFN)	[140]
striped catfish (*Pangasianodon hypophthalmus*)	PA, 25–50 μm	500 mg/kg	28 days	Intestinal damage, hematological abnormalities, decreased survivability, impaired digestion and absorption	[198]
wami tilapia (*Oreochrois urolepis*)	PE, 38–45 µm	1, 10, and 100 MPs/mL	65 days	Small intestinal histopathological changes, reduced growth (final weight, weight gain, total length), impaired digestion and nutrient absorption, altered condition factors	[199]
rainbow trout (*O. mykiss*)	PS, 100–400 µm	10 mg of MPs/fish/day	28 days	No measurable effects on fish intestinal	[200]
European perch (*Perca fluviatilis*)	PLA MPs, 90–150 μm	2% (*w*/*w*) in diet	180 days	Increased reaction to conspecifics; Altered behavior	[201]
African catfish (*Clarias gariepinus*)	LDPE fragments	50 and 500 mg/L	4 days	Biomarker responses; influence physiology	[202]

### 5.4. Adult Fish

Adult fish demonstrate relatively lower vulnerability to MPs compared to those in early life stages, yet they remain susceptible to disruptions in physiological and behavioral processes, often involving oxidative stress, immune alterations, and reproductive impairments [203,204]. These effects vary by fish species, MP type, size, concentration, and exposure duration, with ecological and health implications for aquatic populations (Table 4), though adult fish’s mature systems may mitigate some risks relative to the heightened sensitivity observed in embryos, larvae, and juveniles. In adult fathead minnows (*Pimephales promelas*), environmentally sourced PE-MPs (having size 50–500 μm, and concentration 100–2000 particles/L) reduce growth, lipid reserves, and pigmentation via food dilution, while also disrupting the endocrine system, delaying egg laying, and lowering offspring viability [205]. Adult zebrafish (*D. rerio*) exposed to MPs (PS 0.1–20 μm) exhibit physical and chemical toxicity, including oxidative stress, neurotoxicity, histopathological changes, altered immune gene expression, impaired gill and gastrointestinal integrity, and behavioral disruptions (e.g., daily activity patterns), potentially weakening pathogen defenses and energy metabolism [206,207,208]. Chronic 21-day exposure to PE MPs causes organ-dependent oxidative damage (e.g., changes in GST, GSH, CAT, LPO, SOD), inhibited AChE activity indicating neurotoxicity, and gut microbiota shifts [203,208]. In contrast, adult gilt-head seabream (*Sparus aurata*) ingesting virgin MPs over 45 days (followed by 30-day depuration) showed effective gastrointestinal clearance without stress, growth effects, pathological damage, or long-term accumulation, though 5.3% of livers retained particles, with larger ones occasionally lodging [136]. Generally, MPs in adult fish can impair feeding, foraging, digestion, growth, and immunity, though responses may be neutral for certain processes and less severe than in early stages, highlighting variability in ecological and physiological outcomes [187,209].

## 6. MPs Exposure Mechanisms for Early Life Stages of Fish

MPs may enter the fish body through multiple routes, like ingestion, gill and epithelial uptake, which mostly occurs in the early stages of development [166]. Ingestion of MPs depends on the type of nutrition, with benthic and filter feeding fish being particularly vulnerable, as they make the mistake of consuming MPs resembling plankton or detritus [221]. This vulnerability is exacerbated during the larval and juvenile phases, when smaller body sizes and indiscriminate feeding behaviors increase bioaccumulation risks, potentially leading to compounded effects such as trophic transfer up the food chain and long-term population declines in aquatic ecosystems.

### 6.1. Direct Contamination of Eggs and Larvae

MPs are observed to adhere to the outer membrane of fish eggs, consequently forming physical and chemical barriers that affect the process of oxygen uptake and embryonic growth, and thereby increasing mortality rates [167]. Direct exposure to MPs occurs via surface attachment to fish eggs or ingestion through the digestive tract post-hatching, resulting in health problems such as developmental delays, reduced growth patterns, and compromised reproductive capacity in embryos and juveniles [174]. Larvae may also consume MPs present in the water upon their hatching as they believe it to be food. Consumption may cause their gut to become blocked and thus allow harmful chemicals and other pollutants to enter the body, as well as causing oxidative stress and cell damage and depleting their energy as they grow. Multiple physiological issues and long-term impacts on fish populations and ecosystems have been observed [193,209,222]. In fish, long-term exposure to chemicals in their early life stages may cause them to be less productive in adulthood; they may have fewer eggs or sperm. This undermines the fish population and poses a risk to the strength of water ecosystems [165].

### 6.2. Indirect Exposure Through Contaminated Water and Food

Indirect mechanisms, primarily via polluted water or food sources, represent a key pathway for MPs to enter fish body during the early life stages, inducing harmful effects on physiological growth, reproduction, and health across species [209,210,211,212]. These routes often amplify vulnerabilities in contaminated habitats, with outcomes varying by fish species, life stage, and exposure duration. MPs in polluted water endanger reproductive processes and juvenile maturation by causing endocrine disruptions, which delay egg production, reduce viability, and threaten individual reproductive success and broader fish populations in ecosystems [209,210,211]. Similarly, consumption of contaminated prey or food sources leads to MP accumulation in larvae and juveniles, as evidenced in model species like zebrafish (*D. rerio*) and marine medaka under chronic exposure [223,224,225,226]. These pathways yield comparable effects, emphasizing species-specific vulnerabilities and the urgency of understanding them for sustainable fish conservation.

## 7. Mechanisms of Toxicity

MPs pose substantial toxicological risks to fish, with heightened vulnerability observed in embryonic, larval, and juvenile stages due to their underdeveloped physiological systems, immature detoxification pathways, and ongoing critical developmental processes that render them more susceptible to physical, chemical, and biological disruptions. These mechanisms often involve a cascade of effects, where initial physical interactions escalate into broader physiological impairments, including the adsorption of ancillary contaminants such as persistent organic pollutants (POPs), pesticides, pharmaceutical residues, and heavy metals, which amplify toxicity through bioaccumulation and biomagnification [120,190,203,227,228,229]. Understanding these pathways is essential for assessing long-term ecological consequences on fish populations, aquatic ecosystems, and human food security.

### 7.1. Ingestion and Digestive Blockage

A study revealed that fibers and small pieces of plastics (fragments) are mostly consumed by fish globally [230]. In larvae and juveniles, this results in digestive system blockage, inducing symptoms such as malnutrition, reduced energy availability, and starvation due to phantom satiety, a false sensation of fullness despite insufficient nutrient intake. Severe cases can escalate to intestinal perforations, organ damage, and mortality, as the blocked gut lumen impairs nutrient absorption efficiency and transport functions, ultimately affecting developmental rates, reproductive patterns, and overall survival [227]. Consumed MPs trigger a broad spectrum of responses in fish and other marine organisms. A variety of marine species may consume MPs due to their tiny size. MPs may either be retained in the digestive tract or pass through the stomach if consumed/ingested. If plastic particles were building up in sufficient quantities in the intestines of smaller animals, they may have a similar impact to bigger pieces of detritus and block digestive systems [222,231]. Analysis reveals that the presence of MPs in fish gastrointestinal tracts is transitory and has little potential for accumulation, while transfer to the liver is possible. However, the overall amounts of MPs that will travel through a fish’s digestive system over the course of its lifespan is probably significant and will continue to rise. Similarly, the ingesting of MPs might affect fish health, thus this could be dangerous. Furthermore, ingesting MPs has been shown to cause intestinal obstruction, physical harm, intestinal histological changes, behavioral changes, altered lipid metabolism, and some other consequences [228,229,232]. Consequently, MP ingestion disrupts endocrine functions, leading to reproductive abnormalities, reduced fertility, hormonal interference that hampers egg development and hatch-ability, and immune system degradation through inflammation, heightening vulnerability to pathogens and diseases, with amplified risks in early stages from absorbed contaminants like per- and polyfluoroalkyl substances (PFAS) and pesticides leaching into tissues during gut retention [120,190].

### 7.2. Tissue Damage

MP distribution in zebrafish (*D. rerio*) larvae and juveniles reveals concentrations in intestines, liver, and muscle tissues, with MPs being absent in intestinal tissues but present in hepatic and muscular regions at juvenile stages, indicating stage-specific responses that may lead to long-term organ damage [233]. Larger MPs, including macroplastics, inflict harm through abrasive textures, causing inflammation, tissue damage, and necrosis along the alimentary canal, thereby interfering with organ functionality [234]. In zebrafish (*D. rerio*) gills, MP size dictates toxicity: larger particles induce extensive molecular and oxidative stress, impairing gill function and disrupting osmoregulation, ion regulation, and respiration, which poses significant risks to larval and juvenile breathing capacity and development [233,234].

Circulatory system hazards arise from PS NPs and PE-MPs, which trigger oxidative stress, cell signaling disruptions, endothelial damage, inhibited angiogenesis, and increased thrombus formation, adversely affecting heart function and circulation in early life stages [9]. Even fish exposed to MPs had damage to the hepatic and gut tissue, affect intestinal barrier function, ultimately affecting the growth performance of fish. Kidney health is similarly compromised, with various MPs complications inducing cellular structural damage, immune dysfunction, and overall renal impairment, exacerbated by oxidative stress in zebrafish (*D*. *rerio*) larvae [235,236]. Juvenile tilapia exposed to MPs exhibit concentration-dependent kidney lesions, including capillary congestion, glomerular atrophy, and vacuolation [213]. During the initial development of fish, in the digestive tract, MPs can modify the intestinal mucosa, such as shortening the villi, lamina propria swelling, increasing the number of goblet cells, and so on. These alterations decrease the area of absorptive surfaces and diminish the ability to absorb nutrients [237].

Embryonic and early larval stages face additional risks from MP inhalation, leading to respiratory issues, growth retardation, and physical abnormalities [238]. Surface adhesion on embryos blocks oxygen diffusion, inducing hypoxia, delayed hatching, and developmental defects such as spinal and tail malformations in some species like zebrafish (*D. rerio*) [236,239]. Higher MP concentrations correlate with reduced hatching rates, increased mortality, stunted growth, and amplified developmental issues [240]. Resolving MP pollution is imperative for conserving fish populations and ecosystems.

### 7.3. Oxidative Stress and Inflammatory Responses

MPs directly induce excessive reactive oxygen species (ROS) production, acting as primary cytotoxic agents that damage lipids, proteins, and DNA, disrupting cellular processes and culminating in necrotic cell death. Oxidative stress serves as a key metric for evaluating MP toxicity in models like zebrafish (*D. rerio*). In juvenile loach (15 days post-hatch), polystyrene MPs elevate malondialdehyde (MDA) levels, signifying lipid peroxidation, while activating the Keap1-Nrf2 signaling pathway—comprising Kelch-like ECH-associated protein 1 (Keap1) and Nuclear factor erythroid 2-related factor 2 (Nrf2)—to mediate antioxidant defenses against this complex toxicity [241].

ROS-mediated oxidative stress in MP-exposed fish damages cells, overwhelms protective systems, and impairs normal functions, creating redox imbalances in enzymes like catalase (CAT) and superoxide dismutase (SOD), thereby exacerbating cellular harm [242]. Zebrafish (*D. rerio*) larvae studies show elevated inflammatory and pro-apoptotic markers, including C-reactive protein (CRP), interleukin-6 (IL-6), tumor necrosis factor-alpha (TNF-α), interleukin-1β (IL-1β), interleukin-8 (IL-8), fibrinogen, erythrocyte sedimentation rate (ESR), monocyte chemoattractant protein-1 (MCP-1), and myeloperoxidase (MPO), illustrating the intertwined nature of inflammation and oxidative stress in MP toxicity [242,243]. These effects profoundly impact fish populations and human food supplies, potentially causing substantial declines [243]. Oxidative stress, arising from endogenous and environmental sources, harms biomolecules and cellular structures [244].

In early life stages, MP exposure heightens disease susceptibility via chronic immune suppression and inflammation [245]. Persistent oxidative stress alters immune gene expression, disrupts response balance, and induces immunosuppression, increasing infection risks [180]. For Nile tilapia (*O. niloticus*), combined exposures (e.g., to deltamethrin) elevate liver enzymes, reduce serum proteins, and signal immunological deficiencies [67,213]. This field demands interdisciplinary collaboration to elucidate Keap1-Nrf2 activation, inflammatory upregulation, and long-term physiological/ecological impacts, ultimately informing mitigation strategies for marine habitats and human-reliant species.

### 7.4. Interference with Buoyancy and Mobility

MPs, including microfilms, elevate mortality in fish larvae and juveniles by disrupting buoyancy and locomotion, leading to physiological stress and increased oxygen consumption. Black sea bass (*Centropristis striata*) larvae exhibit heightened oxygen demands post-exposure, compounding stress as buoyancy loss hinders energy maintenance for essential functions [246]. Similarly in medaka larvae, ingested MPs impair swim bladder air regulation, causing swimming deficits and elevated energy costs [191,247]. Furthermore trophic transfer in California grunion (*Leuresthes tenuis*) larvae induces anorexia, delaying growth and survival through energy reserve depletion and structural breakdown [175]. Therefore, MPs compromise energy allocation and stress tolerance, severely hindering early life development. Studies on juvenile European seabass (*D. labrax*) reveal reduced swimming velocity and resistance, impairing navigation, buoyancy control, and ecological roles, leaving individuals vulnerable to threats [184,248]. These multifaceted effects on buoyancy, locomotion, and energy management underscore MPs’ risks to early-stage population survival.

### 7.5. Behavioral Changes

Physical injuries from MPs in aquatic environments profoundly alter fish behavior, particularly swimming patterns, in species such as zebrafish (*D. rerio*), juvenile European seabass (*D. labrax*), and marine medaka (*O. melastigma*) exposed to environmentally relevant concentrations [234,247,248,249]. Affected fish exhibit difficulties including erratic movements, reduced swimming speed, loss of coordination, hyperactivity, diminished social schooling (critical for predator protection and interaction), decreased environmental exploration, and signs of physical exhaustion, all of which impair hunting efficiency, predator escape, navigational skills, and ultimately survival rates and reproductive success [187,211,249].

These behavioral shifts arise from multiple interacting factors: direct physical contact with gills or the digestive tract activates stress responses, elevating hormones like cortisol, which induce cognitive impairments and altered conduct [148]. Neurotoxic effects further aggravate this by disrupting the blood-brain barrier and neurotransmitter activity [148]. Moreover, MPs adsorption of POPs, heavy metals, and polycyclic aromatic hydrocarbons (PAHs) introduces additional toxicants that exacerbate deficits in learning, memory, predator avoidance, and feeding patterns [250]. Collectively, these changes pose severe threats to individual fitness, population stability, and ecosystem dynamics, underscoring the need for comprehensive studies on behavioral ecotoxicology.

### 7.6. Impacts on Feeding and Predator Responses

Fish responses to MP ingestion are modulated by species-specific feeding [222], tendencies and digestive tract morphology, with visually oriented species particularly prone to misidentifying MPs as prey due to visual cues, resulting in feeding disturbances, altered predator-prey dynamics, and ecosystem instability. This misidentification forces consumption of non-nutritive particles, interfering with natural energy intake from authentic food sources and amplifying risks in MP-abundant habitats. For instance, three-spined sticklebacks (*Gasterosteus aculeatus*) rely on visual signals to assess predation threats and adjust foraging, but MPs disrupt this, leading to impaired food acquisition and heightened vulnerability [251]. In goldfish (*C. auratus*) and similar species, MPs and NPs dysregulate appetite through peripheral and central mechanisms, causing energy deficits, gastrointestinal blockage, and internal organ damage that compound the dangers of non-nutritive ingestion [252]. These disruptions extend to broader ecological ramifications, as altered feeding reduces foraging efficiency and predator responses, potentially destabilizing food webs and threatening population sustainability in contaminated aquatic settings.

## 8. Ecological Implications of MPs on Fish and Aquatic Ecosystems

### 8.1. Chemical Toxicity Associated with MPs Pollution

MPs exert chemical toxicity on fish early life stages through the release of inherent additives and the sorption of environmental contaminants, leading to bioaccumulation, physiological disruptions, and long-term ecological consequences [184]. These mechanisms amplify risks during vulnerable developmental periods, where immature detoxification systems and high metabolic rates heighten susceptibility to endocrine disruption, oxidative stress, neurotoxicity, and impaired growth/reproduction, necessitating interdisciplinary research for mitigation strategies in aquatic ecosystems.

#### 8.1.1. Leaching of Additives

It is important to note that MPs are physically polymers, derived from crushed plastics, and many microorganisms lack the enzymes to metabolize these polymers directly. However, low-molecular-weight chemicals inherent in the plastic matrix (i.e., unreacted monomers, initiators, plasticizers, and over 30 types of additives added to enhance product performance) can leach out due to incomplete polymerization and decompose at ambient temperatures [239]. These compounds may contribute significantly to toxicity mechanisms. For instance, MPs ubiquitously release chemical additives, such as plasticizers, flame retardants, and stabilizers, into aquatic environments, posing significant hazards to fish larvae and juveniles by inducing reproductive and developmental toxicities [184]. Phthalates like di(2-ethylhexyl)phthalate (DEHP), a common plasticizer, have been shown in laboratory studies to impair medaka larvae exposed to environmentally relevant concentrations (20–200 μg/L) over 21 days, resulting in stunted growth, altered locomotion, oxidative stress, apoptotic damage, and neurological harm that disrupt essential developmental processes [247,253].

Endocrine-disrupting chemicals (EDCs) leached from MPs, including phthalates and Bisphenol A (BPA), further interfere with hormonal signaling, growth, and development in fish early life stages. BPA, acting as an estrogen mimic, causes endocrine dysfunction in zebrafish (*D. rerio*), leading to abnormal developmental patterns and gene disruptions in thyroid and growth hormone/insulin-like growth factor (GH/IGF) axes, as observed in rainbow trout (*O. mykiss*) eggs via maternal transfer, ultimately impairing larval growth and viability. Flame retardants and similar additives similarly perturb thyroid hormone functions, which are critical for regulating metabolism and development, resulting in delayed maturation, morphological abnormalities, and reduced survival [148,254]. On a broader scale, the leaching of these additives contributes to widespread aquatic ecosystem degradation, manifesting as oxidative stress, endocrine disruption, and hindered growth/development in fish larvae. The ambient decomposition releasing unreacted monomers and initiators adds another layer, potentially facilitating microbial degradation and secondary toxicity. Future research should prioritize this issue through targeted investigations distinguishing polymeric effects from those of leached low-molecular chemicals, as understanding these pathways is essential for assessing long-term ecological consequences on fish populations, aquatic ecosystems, and human food security. Addressing this requires a comprehensive understanding of MP composition, additive migration dynamics, and their synergistic impacts on aquatic biota to inform policies aimed at reducing plastic pollution and chemical contamination in marine and freshwater habitats.

#### 8.1.2. Absorption of Environmental Pollutants by Microplastics

MPs serve as vectors for environmental pollutants, including persistent organic pollutants (POPs), heavy metals, and pesticides (antibiotics), which sorb onto their surfaces and are subsequently ingested by fish larvae and juveniles, triggering oxidative stress, DNA damage, metabolic dysfunction, and enhanced bioavailability [254,255]. Studies on European seabass (*D. labrax*) and zebrafish (*D. rerio*) highlight these risks, with heavy metals like copper and cadmium bound to MPs inducing neurotoxicity, reduced swimming performance, and oxidative stress in larvae [211,248,256]. Polychlorinated biphenyls (PCBs) and polybrominated diphenyl ethers (PBDEs) on MPs further cause immunological and metabolic disruptions, as seen in Atlantic cod (*G. morhua*), where pollutant accumulation leads to epithelial defects, immune deterioration, and potential long-term health impairments [182].

Combined exposures exacerbate toxicity; for instance, MPs and POPs in zebra fish (*D. rerio*) embryos produce physiological and molecular interactions resulting in developmental anomalies, liver damage, and neurological issues [179,257]. MPs enhance the uptake and toxicity of co-pollutants like mercury, with European seabass (*D. labrax*) juveniles showing elevated oxidative stress, neurotoxicity, and lipid peroxidation under MP-mercury co-exposure [211]. Acidic gut conditions in aquatic organisms promote efficient desorption of POPs from MPs, increasing bioavailability and amplifying effects such as oxidative stress and endocrine disruption, which pose amplified threats to early life stages’ survival and ecosystem roles.

### 8.2. Bioaccumulation of MPs in Fish

MPs bioaccumulate in fish across various life stages, with embryos, larvae, and juveniles showing particular susceptibility due to their small body size, elevated metabolic rates, underdeveloped physiological barriers, and immature detoxification mechanisms, which facilitate rapid uptake, tissue distribution, and amplified toxicological risks from both the particles and adsorbed contaminants [258,259,260]. This process extends beyond initial entry points, leading to translocation into vital organs such as the gills, liver, and muscles in diverse species, resulting in chronic effects including developmental abnormalities, reduced organismal fitness, lipid peroxidation, neurotoxicity, stunted growth, and diminished survival rates [259]. Understanding these patterns necessitates a detailed examination of tissue-specific bioaccumulation, where organ-selective retention, influenced by particle size, shape, and polymer type, underscores varying degrees of physiological disruption and ecological risk.

#### 8.2.1. Tissue-Specific Bioaccumulation Patterns

MP bioaccumulation in fish exhibits distinct patterns influenced by particle characteristics (e.g., size, shape, polymer type), species-specific behaviors, and exposure pathways, with implications for organ function, trophic transfer, and human health risks through seafood consumption [230,231,232]. Smaller particles (<100 µm) and fibers often predominate, enabling deeper tissue penetration and exacerbating cellular and systemic toxicities such as oxidative stress and inflammation.

The gastrointestinal tract [261] represents the primary and most common site of MP bioaccumulation in fish, driven by direct ingestion during foraging, where particles are frequently mistaken for natural prey due to visual or olfactory cues [142,259]. Predominant MP forms include filaments and polymers such as PP and PET, as evidenced in Nile tilapia (*O. niloticus*), where MPs accumulate most abundantly in the gastrointestinal tract compared to other tissues like gills and muscles [259]. Freshwater species, including rainbow trout (*O. mykiss*) and common carp (*C. carpio*), are especially prone to this due to their active bottom-foraging habits, leading to high ingestion rates and subsequent disruptions in digestion, nutrient absorption, and gut microbiota balance [142,262]. Chronic gastrointestinal tract accumulation can facilitate the leaching of additives or desorption of sorbed pollutants, amplifying risks of systemic toxicity, energy deficits, and developmental impairments in early life stages, while also serving as a gateway for translocation to other organs [259].

Gills function as a key secondary bioaccumulation site, particularly in filter-feeding or respiratory-active species, where MPs are captured during water filtration or passive uptake, often resulting in mechanical abrasion and functional impairments. In Nile tilapia (*O. niloticus*), gill MPs are typically smaller than those in the gastrointestinal tract and comprise a variety of polymers, including PP, PET, PS, nylon, and PL, reflecting size-selective retention during respiration [263]. Species like Bombay duck (*Harpadon nehereus*), which rely on passive filtration for feeding, accumulate elevated levels of fibrous MPs in gills (averaging 6.98 ± 6.73 MPs/g), posing risks of gill damage, reduced oxygen exchange, ion dysregulation, and oxidative stress [264]. Across multiple fish species, fibers dominate gill accumulations, highlighting this tissue’s role as a frontline barrier that not only traps MPs but also facilitates their entry into the bloodstream, with potential downstream effects on overall health, survival, and human exposure via contaminated seafood.

Muscle tissue displays relatively low MP bioaccumulation compared to other organs, yet it raises significant concerns for food safety and biomagnification due to the potential for NPs translocation and long-term retention [123,265]. In Nile tilapia (*O. niloticus*), muscle MPs are mainly small fragments (<100 µm), suggesting migration from the GIT via epithelial barriers or circulatory transport after prolonged exposure, while NPs are especially concerning as they bioaccumulate in muscles, causing cellular toxicity, inflammation, and bioenergetic disruptions that impair fish fitness and growth [266]. This pattern underscores the human health implications, as edible muscle tissues may serve as vectors for MP transfer in the food chain, necessitating enhanced regulatory monitoring of commercially harvested species in polluted environments.

MPs smaller than 5 µm can translocate from entry sites like the gastrointestinal tract or gills into internal organs via bloodstream circulation, albeit with low absorption efficiency modulated by particle size, composition, and exposure intensity [263]. In fish models, this leads to accumulation in the liver and kidneys, where MPs induce oxidative stress, cellular toxicity, metabolic disturbances, and histopathological changes [255,263]. Planktivorous species ingest MPs through contaminated zooplankton, resulting in gastrointestinal tract buildup that extends to the liver, while pelagic carnivores like Bombay duck (*H. nehereus*) exhibit high overall MP loads (6.98 ± 6.73 MPs/g), dominated by persistent fibers and PET polymers, due to trophic and environmental persistence [146,264,267]. These accumulations trigger health declines, including impaired detoxification, developmental anomalies, and reduced biological performance, with broader ecological ramifications as MPs escalate across trophic levels in food chains.

#### 8.2.2. Pathways of MPs Entry Leading to Bioaccumulation

When evaluating MPs’ possible ecological hazards, it is essential to consider how they bioaccumulate within living organisms. In aquatic ecosystems, MPs are primarily taken up through feeding and respiration in both larval (during the open feeding period) and adult fish [268]. Fish may ingest MPs in numerous ways. After entering the body, they may accumulate in various parts of the body as they are absorbed either directly or through the food chain. The routes are uptake by the gills, oral ingestion (absorbing water-sized particles carried by prey), and skin. MPs are able to accumulate in the digestive tract and can enter the bloodstream. The skin or gills may also absorb some of the small particles. When the particles are in the bloodstream, they are distributed to the gut, liver, gills, kidneys, and muscles. The particles first normally enter through the gut and the gills; then, they are distributed throughout the body depending on the size of the particle, the surface and the size of the fish. Fish in young life stages (larvae, juveniles) tend to contain more MPs than adults, depending on their feeding mode (feeding by filtering or oral intake) [132,260]. These pathways describe the ways in which MPs accumulate and lead to stress in certain tissues, which may have an impact on fish health and their immune system, and they may even be transferred to animals that consume them (higher trophic levels).

##### Microplastic Ingestion Through Water and Sediment

Fish may directly ingest MPs that are suspended in the water or indirectly ingest those deposited in sediments during the feeding period. The exposure route (e.g., dietary or waterborne) and aquatic ecosystem (e.g., freshwater or marine) affect the bioaccumulation of MPs [269]. MP is consumed by fish in water and sediments. They acquire it by ingesting small pieces of plastic in the water, by filtering or preying upon prey with plastic in them, and by chance ingestion via the gills or stomach when taking in sediment or water at the water–sediment interface [270,271] MPs can be adsorbed on sediments and also desorbed. Benthic and reef-associated fish consume MPs as they seek food in or around the sediment or when the particles that had been stirred are resuspended back into the water. The sizes of particles in sediments typically become smaller due to weathering and settlement of organisms on them, and this can increase the possibility of bottom-dwelling species absorbing them [272]. It has been discovered that MPs are present in the stomach, gills, liver, and other organs of various fish species. The location of the plastics and their prevalence are often consistent with fish feeding behavior and habitat locations like in open water or along the sea bed [176,206,263]. MPs are found by fish in the water; in particular, filter feeders or plankton-eating fish are prone to swallowing small particles. For fish that swim in the open water or live at the sea bottom, MPs in the stomach/gut can block the movement of food, reduce their food intake and alter their consumption and energy use. Certain studies also established that minute pieces are transferred to body tissues and result in indications of oxidative stress [198,273].

##### Trophic Transfer via Food Web

MPs bioaccumulate through the food chain, transferring from prey to predator. Trophic transfer is a crucial pathway; MPs also accumulate in fish as they eat prey organisms that have already accumulated or ingested MPs. Some small invertebrates and small fish act as important vectors for MPs, along with the food chain, leading to biomagnification or bioaccumulation in higher trophic ranks [274,275]. For example, the zooplankton in waters of the coastlines store MPs and find their way into fish such as milkfish (*Chanos chanos*) [276].

Trophic transfer is the process by which predators ingest/consume MPs indirectly that have already been ingested by some other prey. Several studies have looked at the trophic transfer of MPs from prey to predators and shown how these trophically available MPs accumulate in the various tissues of predators [274]. Mussel soft tissues were fed to red female blue crabs (*Carcinus maenas*) after being exposed to two sizes of fluorescent PS particles. MPs were transferred from the mussels and gathered in the crabs’ gills, ovary, hepatopancreas, and stomach. The crab hemolymph also contained MPs, which peaked at 0.04% of the exposed MP concentration at 24 h but were nearly eliminated after 21 days of depuration [277]. Copepods were exposed to PE MPs (1–5 μm) to investigate the trophic transfer of MPs from copepods to juvenile seahorses (*Hippocampus reidi*). According to the concentration of these MPs in the copepods, seahorses accumulated them in their stomachs [273]. A study clearly demonstrated that the dietary exposure of mussels (*Brachidontes variabilis*) was the dominant source of MP accumulation in the predatory gastropod mollusk (*Reishia clavigera*). In the experiment, researchers first exposed mysids (*Neomysis species*) to fluorescent PE beads (27–32 μm) at concentrations of 200 and 2000 μg L^−1^ and subsequently fed these mysids to the benthic fish (*Myoxocephalus brandti*). The MP accumulation in these fish was then compared with that in fish directly exposed to MPs in the water. Exposure through the mysid diet resulted in significantly higher—3 to 11 times more—PE accumulation in the fish than direct water exposure, strongly suggesting that trophic transfer is a dominant pathway for fish to accumulate MPs [269].

##### Respiratory Uptake Through Gills

In the respiration process, the banks of the gills draw the suspended MPs in water. Particles may be deposited on the gills or enter the bloodstream. Using the grass carp species (*C. idella*) as a research subject, it was observed that an increase in the rate of water flow resulted in higher rates of accumulation of MPs within the gills and other tissues [278]. The absorption of MPs by gills or the skin can also occur, particularly in huge fish species in the ocean. The route is not normally taken into account but can also lead to bioaccumulation in the presence of large doses of MPs in the water [279,280]. Another possible route of entry of MPs into fish is through drinking of water that has been contaminated with MPs, especially by fish species that consume great quantities of water [279]. MPs of smaller size (less than 0.5 μm) have higher chances of absorption by gill tissues and are systemically distributed [281].

### 8.3. Long-Term Ecological Implications of MPs

The long-term ecological effects of MPs on fish extend beyond individual organisms to influence population dynamics and entire aquatic ecosystems. MPs not only remain in water environments but also accumulate in fish tissues, thereby causing chronic health effects, which affect physiological, behavioral, and reproductive systems.

#### 8.3.1. Sublethal Impacts on the Population Dynamics of Fish

Sublethal MP exposure refers to doses that do not cause immediate death of individual fish but, nevertheless, may have chronic physiological, behavioral, and reproductive effects that accumulate over months, years, or generations. Long-term ingestion or exposure reduces feeding efficiency and nutritional uptake; causes oxidative stress, inflammatory response, poor growth, and changes in behavior (foraging, predator avoidance, migration); and decreases reproductive output and offspring quality, as seen in laboratory work [211,282]. Such malfunctions decrease individual fitness and, when large, can change population vital rates (growth, fecundity, survival) and, consequently, demography and persistence in the long term.

In early life stages, exposure to low levels of MPs in the environment has less impacts on early mortality, but long-term exposure may result in ecological alteration through the presence of sublethal toxic effects and alters the food chain associations. Extensive sublethal harm to biology, as well as food chain interactions, will produce indefinite ecological changes [132].

#### 8.3.2. Biomagnification of MPs and Trophic Transfer

Biomagnification is the process by which pollutants, including MPs and chemicals associated with them, become more and more concentrated as they successfully move up trophic levels of the food web. In contrast to simple bioaccumulation that is limited to a single organism, biomagnification emphasizes an increasing load of the contaminant along the entire food web. MP particles are ubiquitously distributed in aquatic ecosystems, and they are regularly consumed by a wide range of organisms, increasing concerns about their translocation through food webs and possible biomagnification, especially through piscine food chains [283,284].

The biomagnification of MPs and their trophic transfer in fish is a multifaceted problem, which has gained significant interest during recent years. Experimental evidence shows that MP accumulations are heterogeneous due to large and small species of fish and accumulation in various environments, with some showing a scenario of trophic convection and others a highly insignificant or no biomagnification at all [285]. Meanwhile, a scientific study demonstrates that biomagnification causes pollutants including MPs as well as their chemical compounds to grow in concentration through successive levels of the food chain [191].

The absence of biomagnification occurs due to multiple factors including fast expulsion and poor tissue assimilation as well as restricted predator transfer. The selective feeding habits and efficient elimination of organisms result in trophic dilution which causes MPs to decrease in concentration at higher trophic levels [286]. This trophic dilution effect, whereby concentrations of MPs decrease progressively with increasing higher trophic levels, may be explained by the faster rates of excretion and also species-specific feeding habits and elimination processes [286,287]. However, exceptions exist in certain ecosystems or under specific conditions, with a few studies suggesting possible biomagnification in edible fish tissues [286].

Several fish species and other higher-ranking organisms have been shown to exhibit biomagnification. Planktivorous fish were found to contain MPs, which biomagnified into larger predators that preyed on the fish. In the Mediterranean Sea, biomagnifications have been noted in swordfish (*Xiphias gladius*), albacore tuna (*Thunnus alalunga*), and Atlantic bluefin tuna (*Thunnus thynnus*). It was discovered that MPs were trophically transmitted from Atlantic mackerel (*Scomber scombrus*) to grey seal (*Halichoerus grypus*) [132,269].

There is little evaluation of biomagnification of MPs in fresh water. In order to assess biomagnification in the freshwater ecosystem, the MPs in fish of different feeding guilds, such as herbivores, carnivores, and omnivores, were examined. The mean number of MPs in carnivorous and omnivorous fish was 6.09 and 5.85, respectively, being almost equivalent to each other. Omnivorous fish had a higher number of MPs, but it was not relatively higher, while herbivorous fish had 1.88 MPs on average [288]. The number of MPs in freshwater fish trended upward from herbivorous to carnivorous and omnivorous fish. However, it could be because each study was conducted in a different geographic region and each fish consumed a different type of food [288].

Observations in marine ecosystems indicate in several directions that MPs can move from lower to higher trophic levels via food webs, but the extent and strength of the biomagnification of MPs are debated. It has been demonstrated in systematic reviews that a wide range of taxa consume MPs and that they can be passed on to predator species; some studies have shown increases in concentrations of MPs across trophic levels in a specific ecological context [289,290]. Biomagnification of MPs of certain types and dimensions has been observed in some marine food webs, although many studies have reported low or no biomagnification where concentrations are compared across trophic levels. It follows that although the trophic transfer of MPs is a possibility, actual biomagnification, i.e., a progressive increase in concentrations at higher and higher trophic levels, does not emerge as a general occurrence [289]. It is important to note that large-scale syntheses place greater emphasis on bioaccumulation and trophic transfer than universal biomagnification and it is therefore contingent on the nature of the MPs type and ecosystem situation [288,290].

Consequently, additional research in this field is needed. However, investigations into biomagnification and trophic transmission are constrained by methodological inconsistencies, sampling biases, and limited scope, rendering them inadequate for holistic comprehension; thus, future priorities include standardized assessments, broader ecosystem monitoring, and integrative studies to bridge these gaps and inform mitigation strategies.

## 9. Future Perspective

Although the problem of MP contamination of the aquatic environment is well-documented, critical gaps are still present in knowledge of the overall impact on fish and, hence, on human health. It is particularly pronounced for early life stages of fish, which require special concern due to their heightened vulnerability: underdeveloped immune and detoxification systems make them more susceptible to MP-induced oxidative stress, developmental abnormalities, and mortality; limited mobility increases exposure in contaminated nursery habitats like estuaries; and sublethal effects can cascade into reduced reproductive success, threatening population sustainability and ecosystem stability. The main issue is that the movements of MPs within fish tissues and their excretion are not fully understood, which is complicated by the fact that they can bind to other pollutants, such as heavy metals, and are likely to increase their toxicity. This instantly poses pressing concerns regarding the transfer of these polluted MPs to human beings by consumption of fish, presenting a big threat to food safety. This has resulted in a high urgency to thoroughly investigate these internal processes and cumulative health impacts on fish so that sufficient policy and management interventions can be developed to protect threatened fish stocks as well as human health.

## 10. Conclusions

Microplastic pollution impacts the early life stages of fish (embryo, larvae as well as juveniles) because of their permeable biological barriers and incomplete metabolic pathways. MPs interfere with vital developmental functions, causing physical injury (e.g., chorion blockage, organ defects), chemical toxicity via washed away endocrine disruptive toxins, and behavioral defects resulting in decreased survival rates. MPs act as vectors for persistent pollutants, amplifying their ecological impact through bioaccumulation and trophic transfer, thereby threatening entire aquatic food webs. The long-term effects of chronic exposure observed include reduced reproductive performance and loss of population, posing a risk to the stability of the ecosystem and the continued sustainability of fisheries. An international effort is required to mitigate this crisis by reducing plastic waste, advancing better wastewater treatment technologies, and creating stricter regulatory policies. Future studies should focus on understanding the tissue-specific translocation of MPs, the sequential effects along with co-pollutants, and adaptive biomarkers in susceptible organisms. Conservation of the early life stages of fish is not only considered a requirement for aquatic biodiversity but is also important for the maintenance of food security and human health due to the rising trends of plastic pollution.

## Figures and Tables

**Table 2:** Summary of toxicological effects of MPs on larvae stage of various fish species.

Fish Name	MPs size	MPs Concentration	Exposure Time	Effects	Ref.
zebrafish (*D. rerio*)	HDPE MPs, 14.12–120.97 µm	20 mg/L	4 days	Lateral line system damage, morphological damage in GIT	[177]
	PS, 10 µm	10 µg/L	4 days	Developmental delay, biochemical changes	[178]
	PS-NPs, 308.7 ± 77.4 nm	34 μg/L	6 days	Mortality, heart rate and morphological changes	[179]
northern whitefish (*Coregonus peled*)	PS, 2 µm	5–500 µg/L	6 days	MPs accumulation in GIT metabolic, enzymatic disruption, oxidative stress	[176]
inland silverside (*Menidia beryllina*)	Mixed sizes MPs	3.8 µg/L	10 days	Growth retardation and behavior changes	[180]
marine medaka (*O. melastigma*)	PS, 10 μm	2, 20, and 200 μg/L	60 days	Oxidative stress and histological changes; delayed gonad maturation; decreased female fecundity	[181]
Atlantic cod (*Gadus morhua*)	PE, 1–4 µm	13 µg/g	30 days	Pollutant accumulation disrupted skin integrity and immunity	[182]
European seabass (*D. labrax*)	PS, 10–45 μm	0.1, 1, and 10 mg/L	7–43 days	Delayed hatching, reduced growth, increased malformations	[183,184]
Sheepshead minnow (*Cyprinodon variegatus*)	PE, 150–355 µm	50 and 250 mg/L	4 days	Intestinal distention; generated cellular ROS	[185]

**Table 4:** Summary of the toxicological effects of MPs on adult stages of various fish species.

Fish Name	MPs Size	MPs Concentration	Exposure Time	Effect	Ref.
European seabass (*D. labrax*)	Fluorescent MPs, 1–5 μm	0.69 mg/L	30 days	Decreased growth rate, altered feeding behavior, increased cortisol levels	[210,211]
	Mixed MPs	10% MPs in feed	60 days	MPs ingestion cause inflammations in gut, MPs with additives caused potential damage in liver	[212]
Japanese medaka (*O. latipes*)	PS, 200 μm	50–500 μg/L	150 days	No significant impact on growth, development, or survival. Mild alterations in liver observed in 10X group.	[155]
Nile tilapia (*O. niloticus*)	PS beads, 0.1 µm	1, 10, and 100 mg/L	14 days	Potential neurotoxicity, oxidative stress, oxidative damage, Reduced growth, liver inflammation, altered gut microbiota	[213]
zebrafish (*D. rerio*)	PS, 5 µm	20 and 100 μg/L	21 days	Altered gene expression, liver damage, reproductive toxicity, decrease body weight, hepatic glycolipid metabolism disorder	[214,215]
Atlantic cod (*Gadus morhua*)	MPS, 10–90 µm	200 particles/L	30 days	Reduced growth, liver stress, altered metabolism	[216]
marine medaka (*O. melastigma*)	PS, 2, 10, and 200 µm	10 mg/L	60 days	Increase body weight, adipocyte size, hepatic lipid content due to exposure to 200 µm, while exposed to 2 and 10 µm exhibited liver injury and alteration in gut microbiome	[217]
goldfish (*Carassius auratus*)	PS, 0.24 and 8 µm	300 μg/L	7 & 28 days	Inflammations occurred in various organs such as liver and intestine	[218]
	PS, 20 µm	1 mg/L	7 days	Enhanced copper accumulation, oxidative stress, inflammation, apoptosis, and autophagy in hepatopancreas and intestine	[219]
rainbow trout (*O. mykiss*)	PE, 10–300 µm	9800 particles/g feed	14 days	Due to effective excretion, no evidence of translocation of MPs to liver, gonads, after 24 h low MPs found in gut	[220]

## Data Availability

No new data were created or analyzed in this study. Data sharing to this article.
